# Novel Phage Display-Derived Anti-Abrin Antibodies Confer Post-Exposure Protection against Abrin Intoxication

**DOI:** 10.3390/toxins10020080

**Published:** 2018-02-13

**Authors:** Adva Mechaly, Ron Alcalay, Tal Noy-Porat, Eyal Epstein, Yoav Gal, Ohad Mazor

**Affiliations:** 1 Department of Infectious Diseases, Israel Institute for Biological Research, Ness-Ziona 76100, Israel; advam@iibr.gov.il; 2 Department of Biochemistry and Molecular Genetics, Israel Institute for Biological Research, Ness-Ziona 76100, Israel; rona@iibr.gov.il (R.A.); taln@iibr.gov.il (T.N.-P.); yoavg@iibr.gov.il (Y.G.); 3 Department of Biotechnology, Israel Institute for Biological Research, Ness-Ziona 76100, Israel; eyale@iibr.gov.il

**Keywords:** abrin, monoclonal antibodies, phage-display, rabbit, recombinant antibodies

## Abstract

Abrin toxin is a type 2 ribosome inactivating glycoprotein isolated from the seeds of *Abrus precatorius* (jequirity pea). Owing to its high toxicity, relative ease of purification and accessibility, it is considered a biological threat agent. To date, there is no effective post-exposure treatment for abrin poisoning and passive immunization remains the most effective therapy. However, the effectiveness of anti-abrin monoclonal antibodies for post-exposure therapy following abrin intoxication has not been demonstrated. The aim of this study was to isolate high affinity anti-abrin antibodies that possess potent toxin-neutralization capabilities. An immune scFv phage-display library was constructed from an abrin-immunized rabbit and a panel of antibodies (six directed against the A subunit of abrin and four against the B subunit) was isolated and expressed as scFv-Fc antibodies. By pair-wise analysis, we found that these antibodies target five distinct epitopes on the surface of abrin and that antibodies against all these sites can bind the toxin simultaneously. Several of these antibodies (namely, RB9, RB10, RB28 and RB30) conferred high protection against pulmonary intoxication of mice, when administered six hours post exposure to a lethal dose of abrin. The data presented in this study demonstrate for the first time the efficacy of monoclonal antibodies in treatment of mice after pulmonary intoxication with abrin and promote the use of these antibodies, one or several, for post-exposure treatment of abrin intoxication.

## 1. Introduction

Abrin toxin is a 66 kDa type 2 ribosome inactivating glycoprotein (RIP), isolated from the seeds of *Abrus precatorius* (jequirity pea). Owing to its high toxicity, relative ease of purification and accessibility, it is considered a biological threat agent [1]. The toxin consists of two subunits, an enzymatic A-chain (ATA) and a binding B-chain (ATB) linked by a disulfide bridge. The B-chain is a lectin that specifically binds galactose residues at the cell surface, allowing the toxin to be internalized by endocytosis and transported to the endoplasmic reticulum. There, the disulfide bond is reduced, thus facilitating the release of the catalytically-active A-chain into the cytoplasm. Once in the cytoplasm, ATA depurinates a specific adenine residue within a highly conserved stem and loop structure in the 28S ribosomal RNA of the 60S subunit, thereby arresting protein synthesis [2].

To date, there is no effective post-exposure treatment for abrin poisoning and efforts are made to develop an efficient therapy. Passive immunization remains the most effective post-exposure therapy, as was recently demonstrated using a polyclonal fraction of hyper-immune sera isolated from rabbits [3]. Over the last decade, several anti-abrin monoclonal antibodies (mABs) were isolated, several targeting ATA and only one targeting ATB. The mABs were found to be specific and were successfully implemented in several formats of toxin detection [4,5,6,7,8,9,10,11]. The effectiveness of anti-abrin mABs neutralization in vivo was demonstrated only in two cases, where the antibodies were given as a prophylactic treatment [6,8], thus highlighting the need for potent anti-abrin antibodies that will provide effective post-exposure therapy.

We have previously isolated a panel of potent anti-ricin mABs [12] by incorporating immunization methodologies that promote high affinity antibodies in vivo, together with efficient screening methods using phage-display libraries. These antibodies were shown to confer an extended therapeutic window for post-exposure treatment of mice that were exposed to pulmonary intoxication of ricin [13]. We hypothesized that, by applying similar methodologies, we would be able to isolate high-affinity antibodies against both subunits of abrin. Here, we report the immunization strategy and antibody selection procedures taken to reach this end and describe the set of novel anti-abrin antibodies identified.

## 2. Results

### 2.1. Immunization and Characterization of Elicited Antibodies

**Figure 1:**
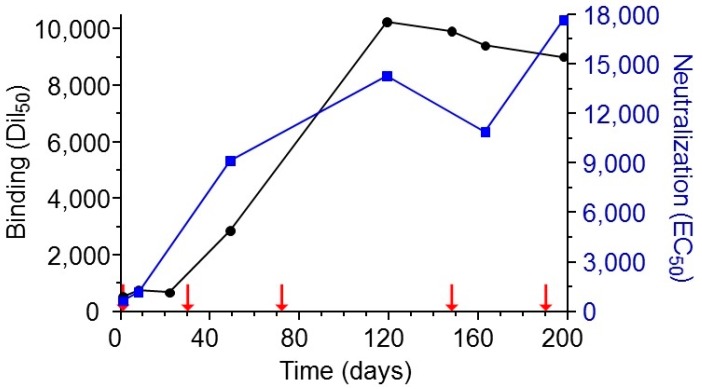
Monitoring of binding and neutralizing anti-abrin polyclonal antibodies development during vaccination. Following initial immunization of rabbit with sub-lethal amounts of abrin, the animal was further immunized using 100 μg of the toxin in incomplete Freund′s adjuvant (indicated by red arrows). Antibody binding titer (black circles) was determined by enzyme linked immunosorbent assay (ELISA) using pure abrin as the coated layer, and the development of neutralizing antibodies (blue squares) was monitored by an in vitro cell assay. Points are of a representative experiment.

The ability to successfully isolate specific and potent antibodies from an “immune library” depends to a large extent upon the effective generation of such antibodies during the immunization process. It is therefore of high importance to characterize the polyclonal antibody response throughout the immunization process and to make sure that optimal titers are reached. Here, the immunization strategy consisted of initial priming of the rabbit with sub-lethal quantities of alum-adhered abrin followed by additional boost injections consisting of high amounts of toxin, mixed with incomplete Freund’s adjuvant. Indeed, over time, a sharp increase in antibody titers was measured with a concomitant increase in their ability to neutralize abrin (Figure 1). By the end of the immunization course, the half dilution value (Dil_50_) corresponding to 50% of the maximal binding of the animal serum towards the coated native toxin was 1:8000–10,000 with a neutralizing titer of 1:18,000.

**Figure 2:**
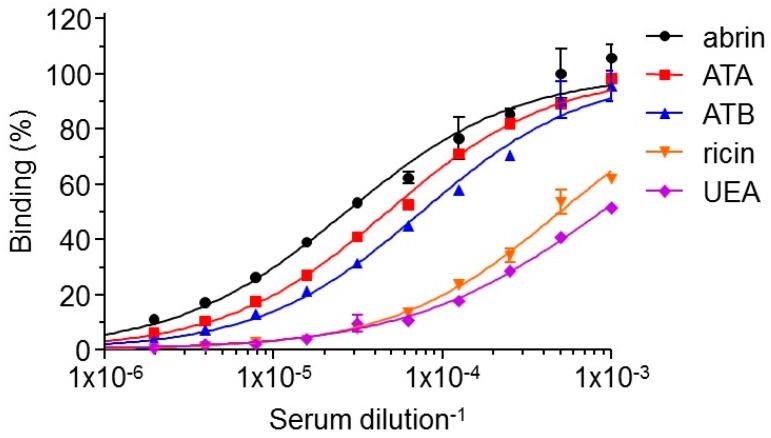
Characterization of the elicited polyclonal anti-abrin antibodies. Abrin, ATA, ATB, ricin or UEA were used as the coating antigens in ELISA to determine the reactivity profile of the antibodies in the sera of the immunized rabbit. Points are the mean ± STD of triplicates.

To determine the specificity of the elicited antibodies, we examined the relative distribution of antibodies recognizing each of the two abrin subunits as compared to the native holotoxin. Interestingly, it was found that the antibody response was equally directed toward both subunits (Figure 2). Abrin contains glycan moieties on both subunits and these plant-associated oligosaccharides are known to be highly immunogenic, therefore could be the target for the elicited antibodies. If a large fraction of the elicited antibodies is indeed directed against the sugar-moieties, it will affect the panning strategy [12] and it was therefore of interest to determine the specificity of these antibodies. To this end, the reactivity of the polyclonal antibodies was tested against two other antigens: (1) ricin, which shares high homology with abrin with respect to its amino acid sequence (40% for the A chain and 96% for the B chain) [14], three dimensional structure [15], activity [16] and sugar-moieties; and (2) *Ulex europaeus* (UEA), a plant lectin [17] which does not share protein-sequence homology with either ricin or abrin, but displays appended glycans, whose composition and structure are the same as ricin and abrin. By comparing the Dil_50_ values for each antigen, it was found that only a small fraction (estimated as about 5%) of the polyclonal antibodies recognize these two antigens (Figure 2), thus indicating that the overall immune response toward abrin is sequence-specific. Taken together, the applied immunization protocol elicited a diverse antibody response which can serve as an excellent source for the isolation of a wide spectrum of monoclonal antibodies directed against abrin.

### 2.2. Isolation of Anti-Abrin Antibodies

A scFv phage-display library was constructed from cDNA templates derived from RNA isolated from the lymphatic organs and peripheral blood of the abrin-immunized rabbit. Based on a literature search of the published primers used to amplify rabbit VH/VL genes, as well as reviewing gene bank databases, a set of degenerate primers was designed to amplify rabbit immunoglobulin families. A total of 16 forward and 7 reverse primers were designed, giving 56 different primer pairs to amplify rabbit VH and Vκ sequences. The VH and Vκ gene pools were assembled by PCR to obtain combinatorial scFv fragments that were inserted into a phagemid vector to create a large, diverse phage-display library. To maximize the possibility of isolating a variety of antibodies targeting different epitopes, the library was subjected to three rounds of panning using two panning strategies: a plate-coated abrin and streptavidin coated beads loaded with biotin-labeled abrin. Following the panning process, individual clones from each panning strategy were screened by direct and indirect (biotinylated abrin adhered to a streptavidin coated plate) phage-ELISA for their ability to bind abrin. In both strategies, more than 80% of the colonies reacted with abrin, and fingerprint analysis of their VH-Vκ genes revealed that all the tested clones (*n* = 17) were unique. To facilitate isolation of clones that target different epitopes, two clones (RB1 and RB3) were reformatted into scFv-Fc antibodies and were used to capture abrin and screen the positive clones in a sandwich ELISA type assay. By the end of the process, 10 clones (Table 1) were chosen for further characterization.

### 2.3. Epitope Binning

**Figure 3:**
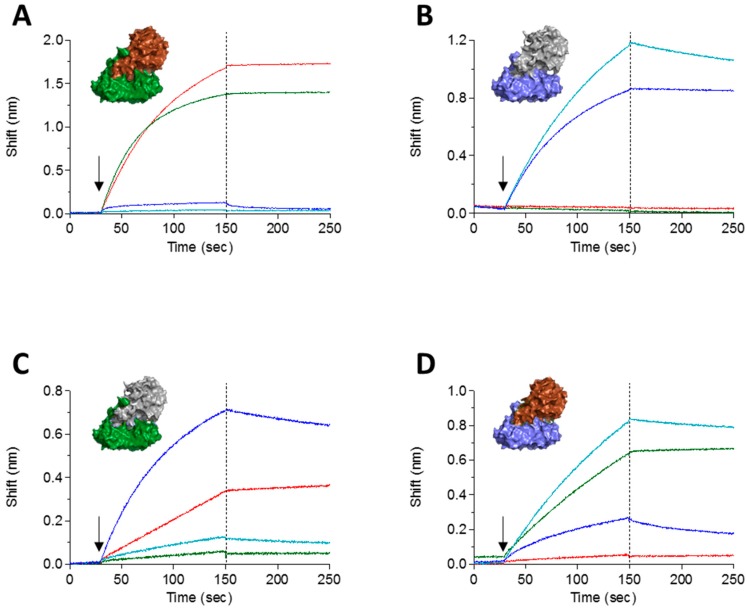
Interaction of anti-abrin and anti-ricin antibodies with abrin-ricin chimeras. The interactions of antibodies with the toxins were measured using Octet Red biolayer interferometry. Biotinylated anti-abrin antibodies RB18 (red) and RB30 (green) or anti-ricin antibodies MH2 (blue) and MH67 (turquoise) were immobilized to the sensors, followed by the addition (indicated by arrow) of: (**A**) abrin; (**B**) ricin; (**C**) chimera of RTA-ATB; and (**D**) chimera of ATA-RTB. The sensors were then immersed in buffer for another 100 s (indicated by dashed line). Crystal structures are of abrin (PDB 1abr; ATA in Brown and ATB in Green), ricin (PDB 2AAI; RTA in Gray and RTB in Blue) and artificially linked RTA-ATB and ATA-RTB.

Chosen clones were reformatted and expressed as chimeric scFv-Fc antibodies [18], composed of the entire rabbit scFv and the IgG1 CH2-CH3 human constant region [19]. As a first step toward classifying the antibody panel according to their targeted epitope, we sought to determine to which abrin-subunit each of the antibodies binds. As the physical separation of toxin subunits might induce structural changes that will hinder the ability of an antibody to bind to its cognate epitope [12], we decided to take another approach. Taking advantage of the fact that the ricin and abrin subunit chains are interchangeable [16], we have created two chimeric toxins: ATA-RTB that consists of the abrin A subunit (ATA) and ricin B subunit (RTB) and RTA-ATB that consists of the ricin A subunit (RTA) and abrin B subunit (ATB). The two chimeric toxins were found to be stable and retain their toxic activity (Gal et al., manuscript in preparation). The binding of anti-abrin antibodies to either ATA-RTB or RTA-ATB was carried out using the Octet Red biolayer interferometry system. To this end, biotinylated antibodies were immobilized on a streptavidin sensor and monitored for their ability to bind each of the chimeric toxins, while the native toxins (abrin and ricin) were used as controls. In Figure 3, the binding curves of two antibodies are presented as representative examples for this assay. Antibody RB18 (red) binds to native abrin (Figure 3A), does not recognize ricin (Figure 3B), binds the ATA-RTB chimera (Figure 3C) and does not bind the RTA-ATB chimera (Figure 3D), thus indicating that this antibody targets the A subunit of abrin. Similarly, it was found that antibody RB30 (green) recognizes the B subunit of abrin (Figure 3). As an internal control, we tested in the same setup two anti-ricin antibodies, MH67 (turquoise) and MH2 (blue) that were previously found to interact with either RTA and RTB, respectively [12]. Indeed, these two antibodies bind the toxin chimeras according to their previously assigned subunit recognition (Figure 3). It should be noted that the binding profiles of all the tested antibodies (either against abrin or against ricin) to their respective toxin-subunits was identical to the binding-profile that was observed when they interacted with the native toxins. Thus, by applying this assay to the antibody panel, it could be concluded that six antibodies are directed against ATA and four against ATB (Table 1). We have also tested the ability of the antibody panel to cross-react with the *Abrus precatorius* agglutinin (APA) using ELISA and found that antibodies RB3, RB10, RB14, RB17, RB28 and RB30 cross react with APA and antibodies RB1, RB6, RB9 and RB18 do not recognize APA.

**Figure 4:**
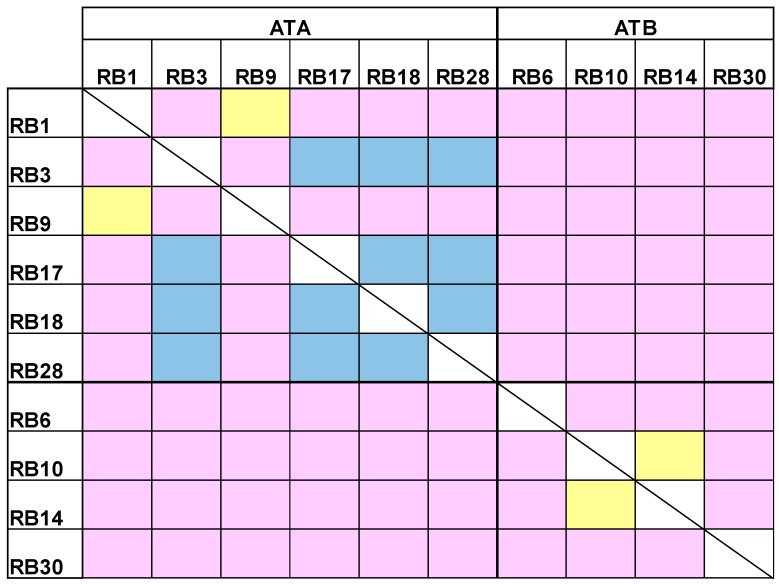
Epitope binning. A complex of the tested antibody and abrin was formed on the Octet sensor and the ability of another antibody to bind this complex was assessed. Yellow: overlapping epitopes; Blue: partially overlapping epitopes; Pink: non-overlapping epitopes.

Our next goal was to cluster the antibody panel according to the binding site of each antibody (epitope binning). For each pair of antibodies, the first antibody was immobilized to an Octet streptavidin sensor, loaded with abrin and further incubated with another antibody. If the second antibody binds simultaneously to abrin, it will form a wavelength shift, thus indicating that the two antibodies bind to non-overlapping epitopes [20]. In contrast, no or a low wavelength shift indicates that the two antibodies bind the same or partially-overlapping epitopes, respectively. It was found that the anti-ATA antibodies can be divided to two groups that represent two distinct epitopes (Figure 4). Interestingly, while antibodies RB1 and RB9 bind to an overlapping epitope, the other anti-ATA antibodies bind to partially-overlapping epitopes, thus suggesting that they are all targeting a larger immunogenic region on the toxin. Out of the anti-ATB antibodies, two (RB10 and RB14) bind the same epitope and the other two bind different and distinct epitopes.

**Figure 5:**
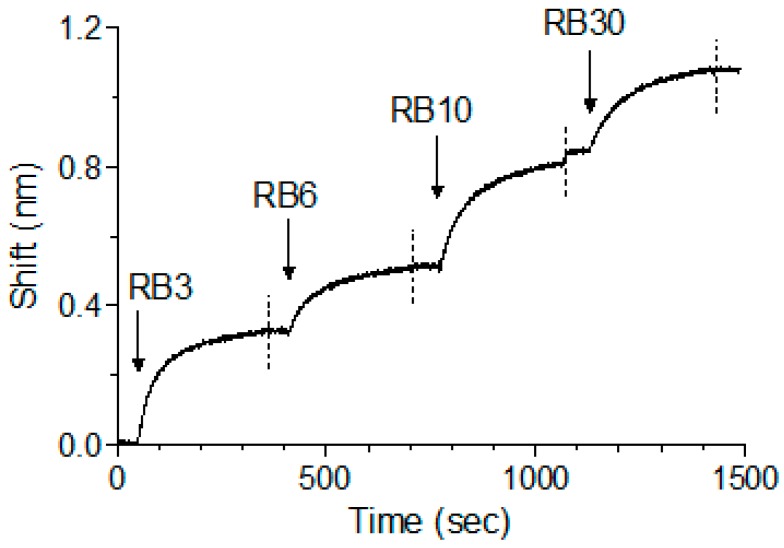
Concomitant binding of anti-abrin antibodies. The ability of different antibodies to bind simultaneously to abrin was tested using Octet Red biolayer interferometry system. RB1 was immobilized to the sensor and loaded with abrin. The formed complex was then immersed in a well containing the indicated antibody (indicated by an arrow), washed again (dashed line) and immersed with the next antibody as indicated.

We have previously shown that, while pairs of anti-ricin antibodies can bind simultaneously, when tested as a cocktail of four, one of the antibodies interfered with the binding of another antibody, probably due to steric interference that resulted from binding of several antibodies to the same toxin [13]. As abrin shares high degree of identity to ricin and since we have identified five distinct epitopes, it was of interest to determine whether the antibodies could bind simultaneously to the toxin. To test that possibility, antibody RB1 was immobilized on the sensor, followed by its immersion in an abrin-containing solution until saturation was reached. The complex was then serially interacted with antibodies RB3, RB6, RB10 and RB30 (as representatives for each epitope group), with a short washing of the formed complex between each step. Surprisingly, and despite the relatively small size of abrin, all five antibodies could bind simultaneously to the toxin with no apparent hindrance (Figure 5).

### 2.4. In Vitro Neutralization of Abrin Cytotoxicity

Next, we asked whether the isolated antibodies possess the capability to neutralize abrin. The first step was to evaluate their activity in an in vitro system that can potentially predict their ability to be effective in vivo. In this assay, the neutralization potency of antibodies to block the toxin from arresting luciferase synthesis in HeLa Ub-FL cells is measured [21]. From a calibration curve, we determined that the abrin concentration needed to inhibit 90% of protein synthesis (IC_90_) is 5 ng/mL (data not shown) and this concentration was set for the neutralization assay. Abrin (5 ng/mL) was incubated with increasing concentrations of the chimeric antibodies, and the mixtures were added to the Ub-FL cells. After 24 h, the residual intracellular luciferase levels were measured, and antibody concentrations needed to neutralize 50% of the abrin activity (ED_50_) were determined. Anti-ATA antibodies RB1 and RB9 (targeting the same epitope) exhibited similar neutralization values (5520 ng/mL and 11,000 ng/mL, respectively; Table 1) with an estimated molar ratio of 1:500–1000 abrin:antibody. Interestingly, antibodies RB3, RB17, and RB18 that target partially overlapping epitopes do not neutralize abrin, while RB28 that overlaps the same epitope presented a neutralizing activity (Table 1). In contrast, all the antibodies that bind to ATB exhibited neutralization activity, with antibody RB30 being the most potent among all anti-abrin antibodies panel (abrin:antibody ratio of 1:70).

### 2.5. Affinity of the Anti-Abrin Antibodies

There is limited data regarding the affinity of anti-abrin monoclonal antibodies in the literature, and so far the reported values were within the nM [11] or high nM range [10]. Moreover, all of these antibodies were directed against ATA. Here, the affinity values of the anti-abrin antibodies panel was determined using the Octet Red biolayer interferometry system. Each antibody was biotinylated, immobilized on the Octet Red sensor and monitored for its abrin binding profile at different concentrations of the toxin (data not shown). The sensorgrams were fitted with a 1:1 binding model, and the association (*k*_on_) and the dissociation (*k*_off_) rate constants were determined (Table 1). In general, all antibodies demonstrated high affinity toward abrin, ranging from 1.5 nM for RB9 and up to a very high affinity value of 20 pM for RB30. Interestingly, while most antibodies binding the same or overlapping epitopes have very similar affinities toward the toxin, antibodies RB1 and RB9 exhibited a tenfold difference (0.1 nM and 1.5 nM, respectively), even though they possess similar neutralizing activity. Taken together, these results demonstrate, for the first time, the isolation of very high affinity antibodies directed against both subunits of abrin.

### 2.6. Post-Exposure Treatment of Abrin Intoxicated Mice

**Figure 6:**
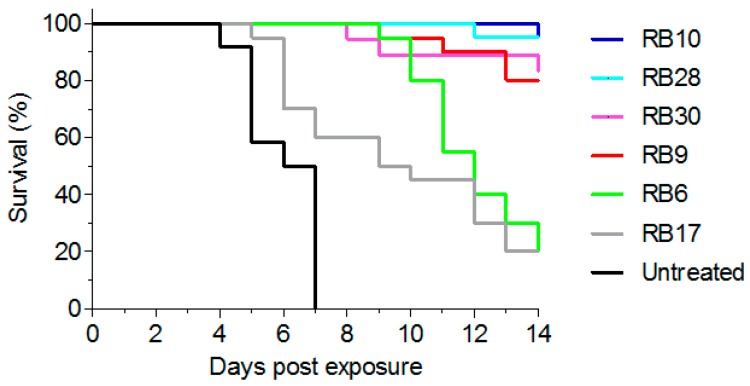
Treatment of abrin-intoxicated mice. Intranasally intoxicated mice (abrin, 10 µg/kg; 2LD_50_) were treated, six hours after intoxication, with intravenous injection of 100 µg of each antibody (*n* = 18 for RB30, *n* = 20 for RB6, RB9 and RB17, *n* = 21 for RB10 and RB28) or left untreated (*n* = 12). Animal survival was monitored for 14 days.

As the major goal of this study was to develop antibodies that will be able to confer post-exposure protection following abrin-intoxication, we tested their activity in vivo. To this end, mice were intranasally exposed to a lethal dose (2LD_50_) of abrin [3] and antibody treatment (100 µg) was given intravenously six hours later. From each epitope group, we selected one representative antibody that exhibited neutralizing capability in vitro. In this model, all untreated animals succumbed within 4–7 days (mean time to death (MTTD) of 6.5 days; Figure 6). Upon treatment, antibodies RB10 and RB28 exhibited high protection (95% survival) with antibodies RB9 and RB30 following closely, with a protection rate of 85% (Figure 6). In contrast, while extending the MTTD of the intoxicated mice to 12 days, treatment with antibody RB6 provided a very low protection rate (20%). In addition, we chose antibody RB17 as another representative of the partially-overlapping group (exhibiting no-neutralizing activity in vitro) and tested its activity in vivo. As would be expected, this antibody provided very low protection ability, even though 20% of the intoxicated mice survived the challenge and the MTTD was somewhat extended to 9.5 days.

## 3. Discussion

By combining a well-designed immunization protocol with advanced methods for the generation and panning of an immunized phage-display library, we isolated a panel of anti-abrin antibodies that target five different epitopes on the toxin surface. These antibodies were found to exhibit high-affinity and specificity toward abrin, and several of them conferred high level of protection to abrin-intoxicated mice when administered six hours post-exposure.

Over the past decade, two attempts were reported with regard to the isolation of anti-abrin antibodies from phage display libraries. The first work employed a naïve human library, from which two specific antibodies were isolated, yet both antibodies exhibited low affinity values (55–110 nM), probably since the human donors were never exposed to this toxin [10]. In another study, Llama derived single domain antibodies were isolated from a phage-display library that was based on amplified genes of animals that were immunized with abrin toxoid [4]. However, due to impurities in the antigen, the derived antibodies were not specific to abrin and recognized either the *Abrus* agglutinin or ricin. Here, the fact that all the isolated antibodies are abrin specific and do not interact with ricin, fit well with the observation that the polyclonal antibody response is highly specific to abrin and a relatively small proportion of the antibodies was raised against the sugar moieties that are common to both abrin and ricin. These results underline the concept that, by characterizing the polyclonal antibody repertoire of the immunized animal, one can predict the outcome of the panning process.

In a previous study, we have isolated a panel of high-affinity antibodies against ricin and found that the ability to neutralize the toxin correlated (for the majority of the antibodies) with their affinity values [12]. In contrast, no such correlation was observed for the anti-abrin antibodies. For example, the antibodies with the highest affinity (RB6 and RB30) displayed lower protective efficiency in vivo when compared to RB10 and RB28 that possess 10-fold lower affinity values. This difference is somewhat surprising in view of the marked similarity between the two toxins; abrin shares a 40–96% amino acid sequence homology with ricin RTA and RTB, respectively and both toxins operate catalytically in the same manner. However, it should be noted that the catalytic performance of abrin is superior to that of ricin [3,22] and that the clinical outcome of pulmonary intoxication with these two toxins is also different [23]. Additionally, the in vitro assay does not entirely recapitulate the antibody activity in vivo. For example, the most potent antibodies in vivo (RB9, RB10, RB28 and RB30) have up to 15-fold differences in their activity in vitro. Moreover, antibody RB6 which demonstrated neutralization activity in vitro has low potency in vivo. In the absence of substantial body of experimental data regarding the mechanism of antibody-mediated neutralization of abrin and the location of neutralizing-epitopes of this toxin, it is difficult to speculate how these antibodies interact and block abrin activity. Moreover, we have identified, for the first time at least five distinct epitopes on the surface of abrin that are spatially distributed, as antibodies against all these sites can bind the toxin simultaneously. We are currently establishing methods to identify the target epitopes of these antibodies, and we believe that their identification will shed light on the unique toxic activity of abrin.

Abrin is classified as a bioterror agent and the pulmonary intoxication route is considered as the most hazardous [24]. However, to date there is no effective post-exposure treatment for abrin poisoning and passive immunization remains the most effective therapy. Despite several efforts to isolate anti-abrin monoclonal neutralizing antibodies, the protection efficacy was so far demonstrated only if antibodies were given as a prophylactic treatment [8,25]. Here, we demonstrate for the first time that the use of monoclonal antibodies for post-exposure treatment of mice that were pulmonary intoxicated with abrin, is feasible. In fact, we have found that several antibodies conferred 85–95% protection rates when administered six hours post exposure. These results are very encouraging and, in the future, elaborated study should be conducted to determine the effectivity of these antibodies when administered at later time points and to define the therapeutic window for efficient treatment against pulmonary abrin intoxication. The fact that our most effective antibodies bind to different epitopes may suggest that they can be applied in combination, to achieve a greater effect.

To conclude, we isolated a panel of high-affinity anti-abrin antibodies that exhibit high protection rates of intoxicated mice when administered six hours post-exposure. The data presented here encourage the use of these novel antibodies, one or several, for post-exposure treatment of abrin intoxication. Moreover, these antibodies can be incorporated in detection systems to promote sensitive identification of the toxin in a variety of matrices.

## 4. Experimental Section

### 4.1. Animal Immunization

Animal studies were approved by the local ethical committee (RB-21-2012, July 2012) and the animals were treated in accordance with regulations outlined in the U.S. Department of Agriculture (USDA) Animal Welfare Act and the conditions specified in the Guide for Care and Use of Laboratory Animals (National Institute of Health, 2011). Pure abrin was prepared as described previously [3]. The purified native abrin holotoxin was used to immunize a female, New Zealand White (NZW) rabbit (Oryctolagus cuniculus). The first four injections consisted of alum-adhered purified abrin that was administered subcutaneously. The first three monthly injections contained 4 µg while the 4th injection, given 10 weeks after the 3rd injection contained 15 µg abrin. Four additional monthly booster injections consisted of 100 µg of purified abrin mixed with incomplete Freund’s adjuvant. Seven days after the last boost, the rabbit was sacrificed, and blood and lymphatic nodes samples were taken.

### 4.2. scFv Library Construction and Screening

RNA extraction from the lymph nodes and blood samples and first-strand cDNA synthesis were carried out essentially as described before [12]. A set of degenerate primers was designed to amplify known sequences of rabbit immunoglobulin families based on published data [26,27,28,29,30,31,32,33,34,35]. A total of 16 forward and 7 reverse primers were designed, giving 56 different primer pairs to amplify rabbit VH and Vκ sequences. A second set of primers was designed, consisting of the same gene specific sequences with additional sequences for linker generation as well as restriction and cloning. The specific primers were utilized for the amplification of VH and Vκ fragments and for the construction of the scFv library exactly as described before [12,36].

Library panning was carried out in two different routes: a solid based route, where the antigen was directly coated on a plate versus a solution based route where the toxin was biotinylated and captured by streptavidin coated magnetic beads. For the solid based route, 10 µg/mL abrin were used to coat a Maxisorp 96-well microtiter plate (Nunc, Sigma-Aldrich, St. Louis, MO, USA). After an overnight incubation the plate was washed and blocked (3% skim milk + 0.05% tween 20 in PBS). Approximately 1 × 10^11^ of blocked phage clones were than incubated for 60 min with the abrin coated plate, followed by one wash with blocker solution and a total of 6 washes with PBST (PBS, 0.05% Tween 20). Bound phages were eluted with 1 mL 100 mM trimethylamine pH 3 (Sigma T0886) for 30 min and the neutralized eluate (in 200 µL 1 M tris pH 7.4) was used to directly infect 5 mL of *E. coli* TG1 strain (Lucigen, Middleton, WI, USA). The plate was than washed again with PBS, and 1 mL of *E. coli* TG1 strain was used to infect the plate directly. Bacterial cultivation and phage packaging were carried out as described earlier [12]. Two additional panning rounds were conducted as described, with the following changes for the 2ed and 3rd rounds respectively: 10^10^ and 10^9^ phage clones were used as input, antigen incubation time was reduced to 30 min and 15 min, blocking buffer was alternated (between 3% BSA to 3% skim milk in PBS) and the PBST washing steps were raised to include 9 or 15 washes with PBST (0.1%). Single colonies were randomly picked from the third panning output, and tested for their binding to abrin.

For the solution based rout, 100 µL streptavidin-coated magnetic beads (Dynabeads M-280, 10 mg/mL, Invitrogen, Carlsbad, CA, USA) were incubated with 300 µg/mL biotin-labeled abrin for 30 min with gentle shaking. The abrin solution was then removed, and the beads were blocked with blocking solution (3% skim milk in PBS) and 1 × 10^11^ phage clones were added for 60. Next, the beads were washed twice with blocking solution, 4 times with PBST (PBS, 0.05% Tween 20) and twice with PBS. Bound phages were eluted as described, and the neutralized eluate (in 200 µL 1 M tris pH 7.4) was used to directly infect 5 mL of TG1. The remaining beads were washed with PBS, re-suspended in 200 µL PBS and used to infect additional 3 mL of TG1. The additional two panning cycles were carried out with the same modifications described for the solid-based rout. 

### 4.3. Fingerprint and Nucleic Acid Analysis

Following colony PCR, 5 µL of the PCR products were taken directly for restriction with 0.5 µL *Mva*I (FastDigest; Thermo Scientific, Waltham, MA, USA) for one hour at 37 °C. The scFvs were sequenced by the ABI Prism 310 Genetic Analyzer (Applied Biosystems, Foster City, CA, USA) using primers TAB-RI and CBD-AS [12].

### 4.4. ELISA

Microtiter plates (maxisorp) were coated overnight with 2 µg/mL of either abrin, ATA, ATB, APA or ricin (for the direct ELISA) or with streptavidin (indirect ELISA) in NaHCO3 buffer (50 mM, pH 9.6), washed and blocked with PBST buffer (0.05% Tween 20, 2% BSA in PBS) for one hour. Serum, individual phage clones, purified antibodies or biotinylated abrin were then added to the plates for one hour, washed and incubated with the detecting antibody (AP conjugated goat anti rabbit (Sigma-A8025), HRP conjugated anti-M13 antibody (GE healthcare, Little Chalfont, UK) or AP-conjugated anti-human IgG (Jackson immunoresearch, West Grove, PA, USA) for scFv-Fc antibodies, as described before [12].

### 4.5. Production of Chimeric Antibodies

The entire scFv was cloned into a mammalian immunoglobulin based expression vector [12] that was modified to contain the human constant CH2-CH3 gene (IgG1), thus resulting in chimeric rabbit-human scFv-Fc antibody. FreeStyle Max 293 cells (Thermo Scientific, Waltham, MA, USA) were transiently transfected with purified plasmid and one week later the antibodies were purified on a HiTrap Protein-A column (GE healthcare, Little Chalfont, UK).

### 4.6. Affinity Measurements, Epitope Binning and Subunit Designation Studies

Binding studies were carried out using the Octet Red system (ForteBio, Version 8.1, Menlo Park, CA, USA, 2015) as described before [12]. Streptavidin-coated biosensors were loaded with biotinylated antibody (5 µg/mL) and then reacted for 300 s with increasing concentrations of abrin. The sensor was then moved to buffer-containing wells for another 600 s (dissociation phase). Sensorgrams were fitted with a 1:1 binding model using the Octet data analysis software 8.1 (Fortebio, Menlo Park, CA, USA, 2015), and the presented values are an average of several repeated measurements. For subunit designation experiments, abrin-ricin chimeras were prepared according to a previously published work [16] with slight modifications (Gal et al., manuscript in preparation). The interaction of antibody-loaded sensors with abrin, ricin and the abrin-ricin chimeras was probed as described. For the binning analysis, antibody-loaded sensors were incubated with a fixed abrin concentration (200 nM) and further interacted with another antibody.

### 4.7. Abrin Neutralization in Vitro Assay

HeLa Ub-FL cells [37] were a kind gift from Professor Piwnica-worms (University of Texas, MD Anderson Cancer Center, Austin, TX, USA).Cells were seeded in 96-well plates (1.5 × 10^4^ cells/well) in medium containing abrin (5 ng/mL) and incubated at 37 °C in the presence or absence of the anti-abrin antibody. Twenty four hours later, cells were resuspended in fresh medium containing MG132 proteazome inhibitor (Sigma C2211 1 µM) for another hour, lysed and the residual intracellular luciferase levels were determined using *D*-luciferin as a substrate and expressed as percent activity determined for untreated cells [21].

### 4.8. In Vivo Protection Assay

Animal studies were approved by the local ethical committee (M-28-2016, March 2016) and the animals were treated in accordance with regulations outlined in the U.S. Department of Agriculture (USDA) Animal Welfare Act and the conditions specified in the Guide for Care and Use of Laboratory Animals (National Institute of Health, 2011). Female outbred ICR mice (Charles River Laboratories, Canterbury, UK) were maintained at 20–22 °C and a relative humidity of 50 ± 10% on a 12-h light/dark cycle, fed with commercial rodent chow (Koffolk Inc., Rancho Santa Fe, CA, USA) and provided with tap water ad libitum. Mice (27–30 g, anesthetized) were intoxicated by intranasal instillation of 2LD_50_ of abrin (10 µg/kg, 50 µL/mice [3] and treated with each mAb six hours after intoxication (*n* = 18–21 for each mAb) or left untreated (*n* = 12). Animals were monitored for 14 days and survival plots were calculated using Prism software (Version 5.01, GraphPad Software Inc., La Jolla, CA, USA, 2007).

## Figures and Tables

**Table 1:** Characteristics of the isolated anti-abrin antibodies.

Antibody	Binding ^a^	ED_50_ (ng/mL) ^b^	*k*_on_ (1/Ms)	*k*_off_ (1/s)	*K*_D_ (nM)
RB1	ATA	5520	4.4 × 10^5^	4.5 × 10^−5^	0.1
RB3	ATA	>100,000	1.2 × 10^5^	6.9 × 10^−5^	0.6
RB6	ATB	5350	3.8 × 10^5^	2.1 × 10^−5^	0.06
RB9	ATA	11,000	2.1 × 10^5^	3.1 × 10^−4^	1.5
RB10	ATB	3800	2.2 × 10^5^	5.9 × 10^−5^	0.3
RB14	ATB	6520	2.2 × 10^5^	1.5 × 10^−4^	0.7
RB17	ATA	>100,000	1.1 × 10^5^	4.9 × 10^−5^	0.5
RB18	ATA	>100,000	1.4 × 10^4^	1.6 × 10^−5^	0.3
RB28	ATA	11,600	2.3 × 10^5^	2.2 × 10^−5^	0.2
RB30	ATB	710	4.4 × 10^5^	7.8 × 10^−6^	0.02

^a^ Binding of each antibody to abrin subunits was determined using the Octet Red system on abrin-ricin subunit chimeras. ^b^ The antibody concentration needed to neutralize 50% (ED_50_) of abrin activity in vitro.
